# Internalization and Viability Studies of Suspended Nanowire Silicon Chips in HeLa Cells

**DOI:** 10.3390/nano10050893

**Published:** 2020-05-07

**Authors:** Sara Durán, Marta Duch, Rodrigo Gómez-Martínez, Marta Fernández-Regúlez, Juan Pablo Agusil, Manuel Reina, Claudia Müller, Álvaro San Paulo, Jaume Esteve, Susana Castel, José A. Plaza

**Affiliations:** 1 Instituto de Microelectrónica de Barcelona, IMB-CNM (CSIC), Campus UAB, Cerdanyola, 08193 Barcelona, Spain; sara.duran@imb-cnm.csic.es (S.D.); marta.duch@imb-cnm.csic.es (M.D.); rogomez@uniandes.edu.co (R.G.-M.); marta.fernandez@imb-cnm.csic.es (M.F.-R.); juanpablo.agusil@imb-cnm.csic.es (J.P.A.); jaume.esteve@imb-cnm.csic.es (J.E.); 2 Departamento de Biología Celular, Fisiología e Inmunología, Facultad de Biología, Universitat de Barcelona, 08028 Barcelona, Spain; mreina@ub.edu (M.R.); cmuller@ub.edu (C.M.); scastel@ub.edu (S.C.); 3 Instituto de Microelectrónica de Madrid, IMM-CNM (CSIC), Isaac Newton 8, Tres Cantos, 28760 Madrid, Spain; alvaro.sanpaulo@csic.es

**Keywords:** silicon, nanowires, cells, biomimetics, microtechnology, microparticles

## Abstract

Micrometer-sized silicon chips have been demonstrated to be cell-internalizable, offering the possibility of introducing in cells even smaller nanoelements for intracellular applications. On the other hand, silicon nanowires on extracellular devices have been widely studied as biosensors or drug delivery systems. Here, we propose the integration of silicon nanowires on cell-internalizable chips in order to combine the functional features of both approaches for advanced intracellular applications. As an initial fundamental study, the cellular uptake in HeLa cells of silicon 3 µm × 3 µm nanowire-based chips with two different morphologies was investigated, and the results were compared with those of non-nanostructured silicon chips. Chip internalization without affecting cell viability was achieved in all cases; however, important cell behavior differences were observed. In particular, the first stage of cell internalization was favored by silicon nanowire interfaces with respect to bulk silicon. In addition, chips were found inside membrane vesicles, and some nanowires seemed to penetrate the cytosol, which opens the door to the development of silicon nanowire chips as future intracellular sensors and drug delivery systems.

## 1. Introduction

Advances in miniaturization technologies are providing new tools to study fundamental issues in cell biology [1,2,3,4]. For instance, integrated devices with nanosized parts offer a great opportunity to develop extracellular sensors for cell mechanics [5] or intracellular devices for biomedical applications such as drug delivery and disease diagnosis [6,7]. On the contrary, micro- and nanoparticles have revealed an enormous potential for intracellular applications, as they are small enough to be internalized by cells. Thus, the capability of silicon technologies to produce nanostructured chips smaller than cells makes silicon a fascinating candidate for intracellular applications [8]. We have previously demonstrated that intracellular silicon chips can be used for single-cell labeling [9,10], biomolecular recognition [11], and cell mechanics studies [12]. On the other hand, devices based on semiconductor nanowires [13,14] have vast potential use in advanced field-effect transistor applications [15,16] and nanomechanics [17,18]. Furthermore, nanowire devices have been proved to transduce chemical and biological binding events into electronics, suggesting their potential for a highly sophisticated interface for biological information [19,20,21,22,23]. Accordingly, extracellular or invasive devices with integrated silicon nanowires have demonstrated their capability in many potential applications in cell biology. As relevant examples, they have been used for delivering biomolecules into living cells by using the ability of vertical silicon nanowires to penetrate the cell membrane [6,7], as efficient captors of circulating tumor cells, as shown by nanopillar arrays [24,25], or for localized single-cell electroporation [26]. Furthermore, it has been reported that individual silicon nanowires can be internalized by cells [27,28]. However, the small size of single nanowires limits their prospective application inside living cells due to their difficult intracellular visualization, their small surface area for molecular delivery, and the difficulty to implement a transduction principle for sensing applications. In contrast, silicon nanowires integrated on planar silicon chips have been demonstrated to offer innovative possibilities for a wide range of applications [29]. Hence, in this work, we propose that silicon nanowires, used as building blocks integrated on cell-internalizable silicon microchips, will open new application opportunities of intracellular biology.

## 2. Materials and Methods

### 2.1. Technology for the Fabrication of Microchips Decorated with Silicon Nanowires and Isolated Silicon Nanowire Entangled Meshes

**Figure 1:**
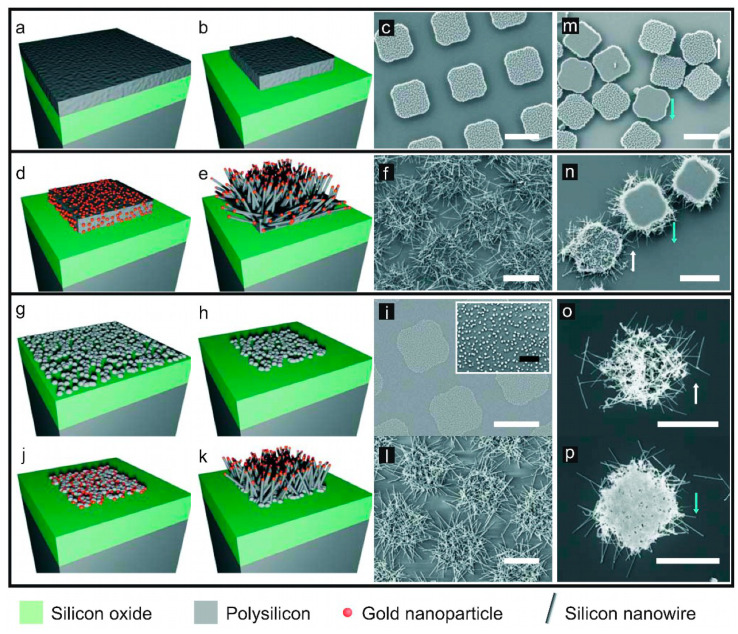
Fabrication of the silicon chips. Polysilicon microchips (SiµCs), (**a**) deposition of a 500 nm-thick polysilicon layer on a silicon oxide sacrificial layer; (**b**) Polysilicon patterning delimited the device; (**c**) SEM image of the fabricated devices on the wafer. Polysilicon microchips decorated with randomly distributed silicon nanowires (SiµC+SiNWs), (**d**) selective gold nanoparticle deposition on SiµCs; (**e**) Silicon nanowire growth via the vapor–liquid–solid (VLS) mechanism; (**f**) SEM image of the fabricated devices on the wafer. Silicon nanowire entangled meshes (SiNW-Meshes), (**g**) Polysilicon deposition on the silicon oxide sacrificial layer was stopped at the nucleation stage, and a non-continuous polysilicon layer was formed; (**h**) The polysilicon nanoclusters patterning shapes the device; (**i**) SEM image of polysilicon nanoclusters (inset, image zoom); (**j**) Selective gold nanoparticle deposition on the nanoclusters; (**k**) Silicon nanowire growth via the VLS mechanism; (**l**) SEM image of the fabricated SiNW-Meshes on the wafer; (**m**) SiµCs; (**n**) SiµC+SiNWs; and (**o**,**p**) SiNW-Meshes after their release. Arrows indicate the chip orientation (white: top-side up, blue: bottom-side up). Scale bar = 3 µm. White scale bars = 3 µm. Black scale bar = 500 nm.

Correlated technical approaches were followed to fabricate three distinct silicon microchips with well-controlled dimensions and different morphologies: (1) polysilicon microchips (SiµCs) to be used as a control during the biological experiments; (2) polysilicon microchips decorated with randomly distributed silicon nanowires (SiµC+SiNWs), and (3) isolated silicon nanowire entangled meshes (SiNW-Meshes). The starting substrate for all silicon microchips consisted of a 1 µm-thick silicon oxide layer deposited by chemical vapor deposition (CVD) over a 100 mm-diameter p-type silicon wafer (Figure S1a). SiµCs were produced out of a 500 nm-thick polysilicon layer deposited by chemical vapor deposition (Figure 1a and Figure S1b) over the previous oxide layer. Then, a 1.2 µm-thick HiPR 6512 photoresist (Fujifilm, Valhalla, NY, USA) was spun onto the polysilicon layer to subsequently define the lateral dimensions by a photolithographic step followed by polysilicon dry etching (Figure 1b and Figure S1c–f). Finally, the photoresist was stripped, obtaining an array of 3 µm × 3 µm chips separated by 3 µm (Figure 1c). SiµC+SiNWs were produced by the initial deposition of gold catalyst nanoparticles over a batch of SiµCs using the galvanic displacement deposition method, ensuring nanoparticle deposition only at the polysilicon surfaces of the microchips (Figure 1d and Figure S1h) [30]. Then, the nanowires were grown using the vapor–liquid–solid mechanism (VLS) (Figure 1e) [13]. Randomly oriented nanowires were obtained, as polysilicon is composed of silicon crystals, e.g., grains, which have random crystallographic orientations (Figure 1f and Figure S2). Lastly, SiNW-Meshes were produced by shortening the polysilicon deposition time in order to obtain isolated nucleated polysilicon nanoclusters (Figure 1g). The diameter and density of the nanoclusters were fixed by the deposition conditions (temperature 580 °C, pressure 350 mTorr, and a SiH_4_ flow rate of 40 sccm). The nucleation of nanometer-scale crystallites occurs in the initial stage of polysilicon deposition [31], which would form a continuous layer if the process continues (SI, Figure S2). After nanocluster formation, a photolithographic step followed by polysilicon dry etching (Figure 1h,i) defined the lateral dimensions of the chips (3 µm × 3 µm). Again, the galvanic displacement deposition of gold nanoparticles ensured their deposition only on the polysilicon nanoclusters (Figure 1j). Finally, the silicon nanowires were grown (Figure 1k), and entangled meshes were obtained due to the random crystallographic orientations of the nanoclusters (Figure 1l).

Lastly, the chips were released by sacrificial etching of the silicon oxide layer in vapors of 49% HF, then suspended in 96% ethanol by using an ultrasonic bath, centrifuged at 5000× *g* for 3 min (MiniSpin Plus^®^, Eppendorf AG, Hamburg, Germany), and collected in Eppendorf tubes for later cell studies (Figure 1m–p). Due to the fragility of the SiNW-Meshes, the process had to be slightly modified, softening the release conditions, avoiding the application of ultrasounds, and reducing the spin time to 2 min.

### 2.2. Nanowire Growth Method Assisted by Catalyst Gold Deposition

Gold nanoparticles were selectively deposited on the polysilicon chip surfaces via galvanic displacement by immersing the substrates in a reverse micellar microemulsion. The microemulsion was synthesized by mixing an aqueous plating solution with n-heptane and the surfactant sodium bis(2-ethylhexyl) sulfosuccinate (AOT, C_20_H_37_O_7_SNa). The aqueous solution consisted of 0.2 M HF and 0.01 M KAuCl_4_, while the AOT/heptane solution contained a final concentration of 0.33 M AOT in n-heptane. Micellar radius (R_m_) was determined by the water-to-surfactant molar ratio, R = [H_2_O]/[AOT], according to the empiric law R_m_ = 0.175R + 1.5 [30]. A molar ratio of R = 20 and an immersion time of 10–30 s were used. Silicon nanowires were grown via the VLS mechanism in a homemade CVD system at 750–800 °C and atmospheric pressure. We used 10% H_2_ in Ar as both diluent and carrier gas, with flows rates of 270 sccm and 40–50 sccm, respectively. The carrier gas was passed through a liquid SiCl_4_ bubbler kept at 0 °C to maintain a constant vapor pressure.

### 2.3. Cell Culture and Silicon Chips Internalization Methodologies

HeLa cells were cultured in Dulbecco’s modified Eagle medium (DMEM) containing 1 g/L glucose and supplemented with 10% fetal calf serum, 2 mM glutamine, 5 U/mL penicillin, and 5 µg/mL streptomycin. The cultures were maintained at 37 °C in a 5% CO_2_–95% air atmosphere. Upon reaching 80% confluency, the cells were detached using a 0.25 mg/mL trypsin/EDTA solution and seeded at a density of 50,000 cells/cm^2^ in the specified support for each experiment. After 24 h, the medium was replaced by an internalization solution containing silicon chips (SiµCs, SiµC+SiNWs, or SiNW-Meshes), in a ratio of 10 silicon chips/cell, and FuGene^®^ 6 (Promega, Madison, WI, USA) in Opti-MEM medium (Thermo Fisher Scientific, Waltham, MA, USA). After 24 h, the internalization solution was removed, and the cells were washed for three times with Opti-MEM and processed for observation.

### 2.4. Living Cells Confocal Microscopy and Viability Assay

To assess cell viability, the membrane-permeable dye Calcein acetoxymethyl ester (Calcein-AM, Invitrogen, Carlsbad, CA, United States) was prepared as a stock solution of 2 mM in dimethylsulfoxide, stored at −20 °C, and used at the final concentration of 5 µM in Opti-MEM. Cells seeded in 8-wells Nunc^®^ Lab-Tek^®^ II (Thermo Fisher Scientific, Waltham, MA, USA) chambered coverglass and incubated with the internalization solution as described above were rinsed with Opti-MEM and incubated with 50 µL of Calcein-AM working solution at 37 °C in a CO_2_ atmosphere. After 1 h, the Calcein-AM solution was removed, and the cells were washed three times. The cells were maintained for 20 min at room temperature for a complete de-esterification of the dye prior to their observation. Calcein-AM is a non-fluorescent hydrophobic compound that easily permeates intact live cells. The hydrolysis of Calcein-AM by intracellular esterases produces calcein, a hydrophilic. strongly fluorescent compound that is well retained in the cell cytoplasm and that can be measured as a viability indicator. Images for quantification were obtained using the Confocal Laser Scanning Microscope (CLSM) Leica TCS SP2 (Leica Lasertechnik GmbH, Mannheim, Germany) adapted to an inverted Leitz DMIRBE microscope and using a HC Plan Apochromatic ×20/0.70 oil immersion objective. To obtain the fluorescence images from calcein, cells were excited with the 488 nm line of an argon ion laser, and the emission light of 500–600 nm was acquired. Silicon chips were simultaneously localized with the reflection mode of the same excitation line in each image. Fluorescence density of calcein was measured using ImageJ software (v1.53a, NIH, Bethesda, MD, USA, http://imagej.nih.gov/ij/). Regions of interest were set for each cell, and the presence or absence of silicon chips in the cells was determined by the observation of dark spots and checked with the refection-mode image of each section. Experiments were performed in triplicate, and at least 20 cells for each condition were measured in each experiment.

### 2.5. Correlative Light and Electron Microscopy of Silicon Chips in HeLa Cells

HeLa cells were plated on square, home-made gridded glass pieces (1 cm × 1 cm) and incubated with the internalization solution as described previously. Later, the samples were fixed with 2.5% glutaraldehyde (EM grade; Sigma-Aldrich, St. Louis, MO, USA), dehydrated through a graded series of ethanol/water mixtures up to 100% ethanol, and dried by the critical point method. The initial localization of the silicon chips was made by bright-field optical microscopy (BFOM), using an Eclipse ME600 upright optical microscope (Nikon Minato City, Tokyo, Japan) at ×100 magnification (0.8 NA LU Plan ELWD 3.5). Images were recorded on an 8-bit color CCD camera (DXM1200F, Nikon, Minato City, Tokyo, Japan). The cells of interest were mapped on the gridded glass pieces and further localized and observed by Scanning Electron Microscopy (SEM) at 5 keV (Auriga, Carl Zeiss GmbH, Oberkochen, Germany). Once observed by SEM, the same cells were studied by Focused Ion Beam–Scanning Electron Microscopy (FIB–SEM) using Ga^+^ ions in a dual beam instrument (1560XB Cross Beam, Carl Zeiss GmbH, Oberkochen, Germany).

### 2.6. EDX Analysis of Chips and HeLa Cells

HeLa cells seeded in 60 mm-diameter dishes and incubated with the internalization solution as described above were fixed with 2.5% (v/v) glutaraldehyde in 100 mM phosphate buffer (PB, pH 7.0) for 1.5 h at 4 °C. The fixed cells were harvested and pelleted, followed by three washes with 100 mM PB. The cells were then post-fixed in 1% (w/v) osmium tetroxide for 2 h at 4 °C and washed three more times with 100 mM PB. The cells were dehydrated through a graded acetone series, resin-infiltrated at room temperature with several increasing Epon12/acetone mixtures and, finally, embedded in fresh Epon12 resin (EMS, Hatfield, PA, USA) for 5 h and polymerized for 48 h at 60 °C. A Reichert ultramicrotome was used to produce 150 nm-thick slices that were collected over Formvar-carbonated copper grids and stained with uranyl acetate (7%) and lead citrate. Energy dispersive-X-ray (EDX) analysis was performed on cell pellets sections. Images were captured by an INCAx-act SEM (Oxford Instruments, Abingdon-on-Thames, Oxford, UK), and the EDX spectrums were obtained using a PentaFET-Precision system (Oxford Instruments, Abingdon-on-Thames, Oxford, UK) attached to the SEM equipment.

### 2.7. Sedimentation Tests of the Silicon Chips

The SiµCs, SiµC+SiNWs, and SiNW-Meshes were suspended by ultrasounds in 96% ethanol, centrifuged at 5000 g for 3 min (MiniSpin Plus^®^, Eppendorf AG, Hamburg, Germany), and collected in three Eppendorf tubes, respectively. Next, the three samples were pipetted, and 2 µL drops were deposited on a clean silicon substrate. After the solvent evaporated, the substrates were observed by SEM. The chips on the substrates were counted in a top-side up and a bottom-side up position and analyzed using ImageJ software. For each type of devices, three sets of 100 devices were counted for statistics.

## 3. Results

### 3.1. Device Fabrication and Morphological Studies

We fabricated silicon chips with three different morphologies in order to study their cell internalization and viability: SiµCs, SiµC+SiNWs, and SiNW-Meshes. All chips were fabricated using semiconductor technologies based on photolithography to control their dimensions, in combination with bottom-up silicon nanowire growth for high-aspect-ratio nanostructures (Figure 1 and Figure S1). SEM imaging was used to study nanowire morphology (length, width, and density), which depends on the growth parameters. The density and morphological features of the silicon nanowires are shown in Table 1 and Figure S3. Nanowire growth times were fixed to 60 s and 90 s for SiµC+SiNWs and SiNW-Meshes, respectively. A longer growth time was selected for the meshes in order to obtain longer wires and ensure nanowire entanglement, as, in this case, there was no 500 nm-thick polysilicon platform (SiµCs) to ensure the structural integrity of the chips. An excessive wire growth time can ruin chip collection, as nanowire entanglements between neighboring chips can form (Figure S4). Lastly, the chips were released by sacrificial etching of the silicon oxide layer in vapors of 49% HF (Figure 1m–p), suspended in 96% ethanol, centrifuged at 5000 rpm for 3 min (MiniSpin Plus^®^, Eppendorf AG, Hamburg, Germany), and collected in Eppendorf tubes for posterior cell studies. Top-side-up and bottom-side-up images of SiµCs did not reveal any relevant morphological difference between them (Figure 1m). On the contrary, SiµC+SiNWs showed larger differences (Figure 1n), as only the top side showed nanostructured silicon nanowires. On the other hand, SiNW-Meshes showed different nanostructured morphologies on the two sides, i.e., polysilicon nanoclusters on the bottom-side-up and silicon nanowires on the right-side-up (Figure 1o,p).

### 3.2. Cell Viability Assays

**Figure 2:**
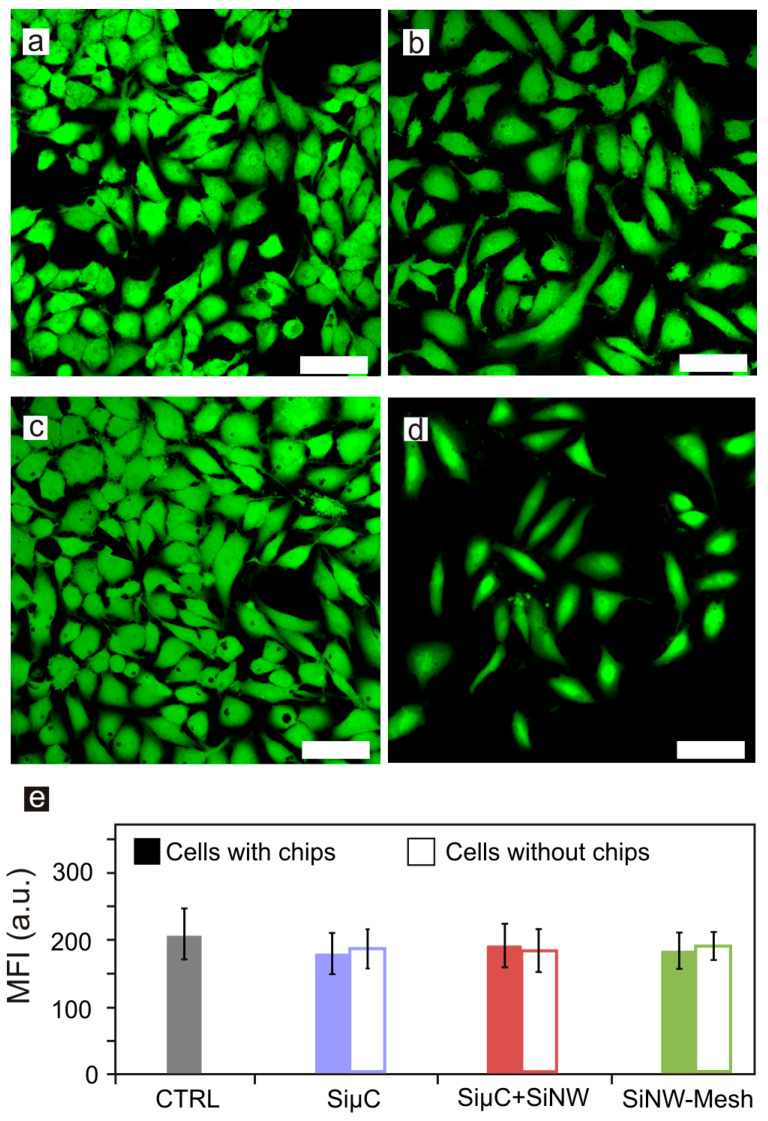
Cell viability assays. Confocal images of (**a**) control cells and cells with internalized (**b**) SiµCs, (**c**) SiµC+SiNWs, and (**d**) SiNW-Meshes. (**e**) Graph of HeLa cells viability measured as the mean fluorescence intensity (MFI) obtained by the Calcein method. Cells count, n, for (e): Control n = 209, SiµCs n = 366, SiµC+SiNWs n = 377, SiNW-Meshes n = 375. Scale bar = 50 µm.

Our previous works demonstrated that lipofection of SiµCs and silicon-based pressure sensors with similar dimensions was possible [11,12]. Thus, in this work we evaluated the lipofection of chips with integrated silicon nanostructures. HeLa cells were lipofected with FuGene^®^ and the fabricated chips (see Materials and Methods). Later, HeLa cells viability was confirmed after 24 h exposure to SiµCs, SiµC+SiNWs, and SiNW-Meshes using the Calcein–AM method. Images related to the viability studies were obtained using a Confocal Laser Scanning Microscope (CLSM) (Figure 2a–d). Microchips appeared as black areas inside the cells. Remarkably, as shown in Figure 2e, the viability of the cells with internalized silicon chips was practically unaffected.

### 3.3. Chip Internalization Studies in HeLa Cells

**Figure 3:**
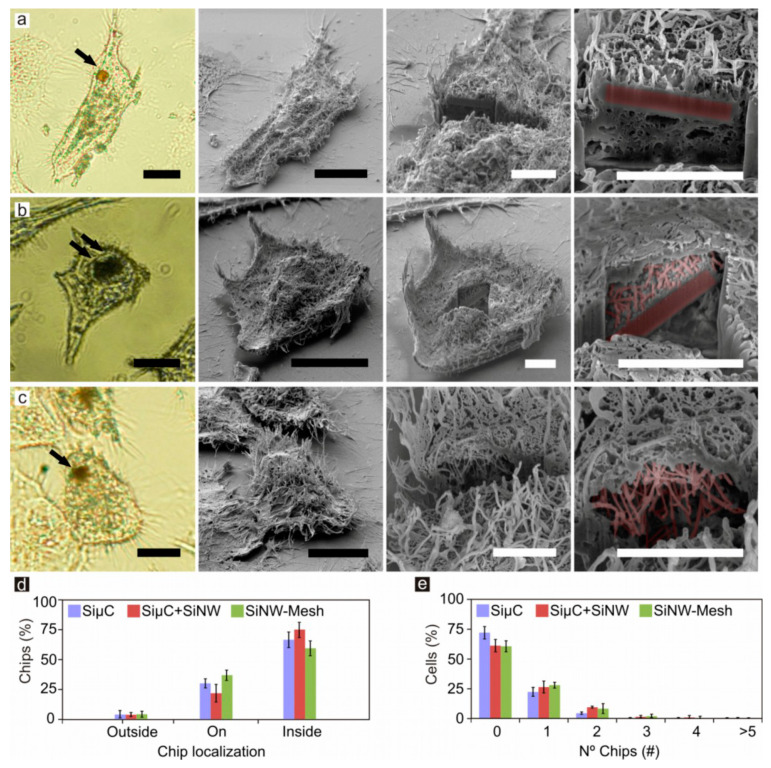
Chip internalization in HeLa cells. HeLa cells with internalized (**a**) SiµC, (**b**) SiµC+SiNWs, and (**c**) SiNW-Mesh. Panels from left to right show: bright-field optical microscopy (BFOM), SEM, SEM after focused ion beam (FIB) milling, and pseudo-colored detailed image (pink color indicates silicon). Black arrows point out chips’ position. (**d**) Graph showing the proportion of chips laying on the substrate outside the cells, on the cell membrane, and inside cells. (**e**) Graph showing the proportion of cells with internalized chips. Chips and cells counts, n, for (**d**,**e**): SiµCs n = 170 and cells n = 345, SiµC+SiNWs n = 360 and cells n = 524, SiNW-Meshes n = 297 and cells n = 418. Error bars: ± 1SD. Black scale bars = 10 µm. White scale bars = 3 µm.

We next analyzed chips cellular uptake by using a BFOM. The chips appeared as opaque areas in BFOM images because of the lower optical transmission of silicon (Figure 3a–c, left column). The information provided by these images was not enough to unambiguously determine whether the chips were inside or outside the cells. For this reason, optical and SEM correlative inspections and a detailed cell mapping were combined to confirm chip internalization. SEM inspection only revealed chips partially or totally outside the cells and did not allow the detection of those chips which were totally internalized inside the cells (Figure 3a–c, second column from the left). Thus, complementary focused ion beam (FIB) milling (S5 and Materials and Methods) was performed to visualize the chips that were completely internalized and preserved inside the cells. Although internalization depends on particle morphology and shape [32], we found chip internalization in HeLa cells for the three different morphologies (Figure 3a–c, two right columns). Then, the localization of chips in the cell cultures was analyzed. The majority of the chips for the three morphologies was cell-internalized at levels between 59.2% and 74.8% (Figure 3d), and a lower proportion (21.6–36.7%) was partially internalized or adherent to the cell membrane, indicative of an initial stage of internalization. Although a large population of the cells, ~25%, with internalized chips carried only one chip (Figure 3e), cells carrying up to 6 chips were found. These results show a great cell capacity to internalize silicon chips, and, remarkably, no significant differences with respect to internalization were found between chips with micro- and nanomorphologies.

**Figure 4:**
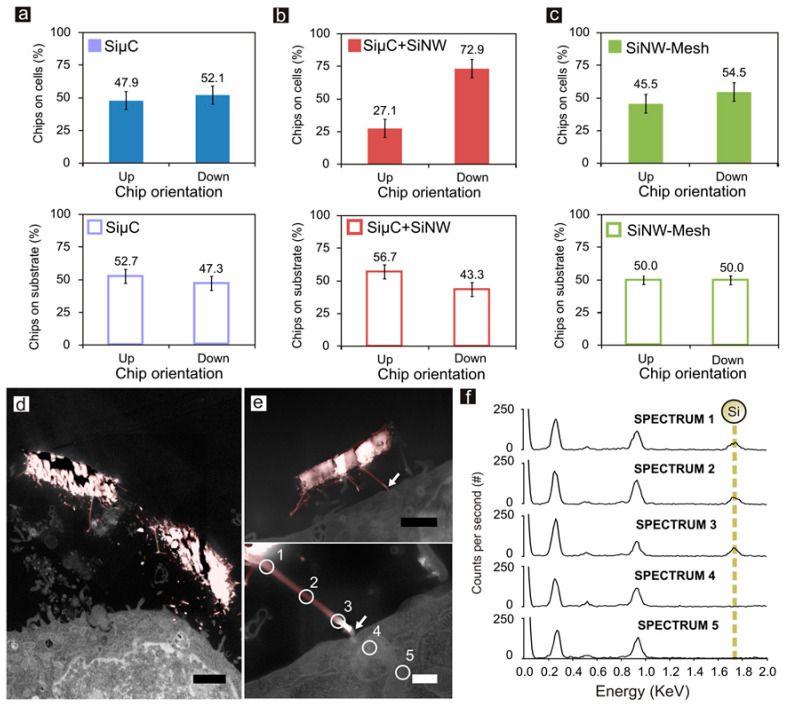
Silicon morphology-dependent orientation at the initial stage of internalization. (**a**–**c**) Graphs showing the proportion of chips on the cell membrane (top panels) and on the silicon substrate after the sedimentation tests (bottom panels) with the topside facing upwards or downwards for SiµCs, SiµC+SiNWs and SiNW-Meshes, respectively. Pseudo-colored SEM images showing the initial contact of (**d**) two and (**e**) one SiµC+SiNWs chips with a HeLa cell (pink color indicates silicon); bottom panel, detailed image of a nanowire on the cell membrane. White arrows indicate nanowire–cell contact sites. White circles indicate EDX spectrum points. (**f**) EDX spectra confirmed silicon presence. Chips count, n, for (a-c, top panel): SiµCs n = 170, SiµC+SiNWs n = 360, SiNW-Meshes n = 297. Chips count, n, for (a-c, bottom panel): SiµCs count n = 300, SiµC+SiNWs n = 300, SiNW-Meshes n = 300. Error bars: ±1SD. Black scale bars = 1 µm. White scale bar = 200 nm.

We also explored the initial stage of chip internalization. The top and bottom sides of SiµCs have equivalent morphologies, and, consistently, the SEM images did not reveal any preferred chip orientation, top-side-up or bottom-side-up (Figure 4a, top panel). On the contrary, the strong morphological asymmetry of SiµC+SiNWs resulted in a favored orientation, which corresponded to that of the contact of the nanowire with the cell membrane (Figure 4b, top panel). Finally, SiNW-Meshes did not present statistical differences in chip orientation during cell internalization, in agreement with the small morphological differences between the top and bottom nanostructured sides (Figure 4c, top panel). These results indicate that initial cell contact was facilitated by nano-structured silicon (Figure 4d,e) with respect to micro-structured silicon. In doubtful cases, where it was difficult to distinguish cell filopodia of silicon nanowires, energy-dispersive X-ray spectroscopy (EDX) was used for material identification (Figure 4f and Figure S5 and Materials and Methods). Sedimentation tests only with silicon chips were carried out in ethanol to discard any fluid dynamic effects on chip orientation during deposition (Figure 4a–c, bottom panels and Materials and Methods). Although ethanol allowed a faster dissipation and final clean surface for characterization, exhaustive sedimentation studies should be done in culture medium. Significant differences were observed only for SiµC+SiNWs (Figure 4b, bottom panel). Regardless of their initial orientation, it seems that the chips settled down with the flat micropart turned down and the nanowire side turned up and remained in this orientation, similar to that of a shuttlecock.

**Figure 5:**
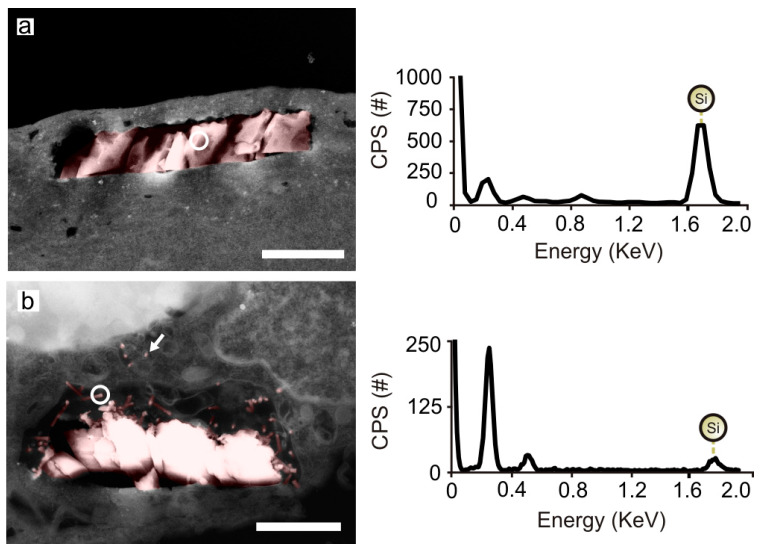
Silicon chips within HeLa cell compartments. Pseudo-colored images (left panels) showing internalized (**a**) SiµC and (**b**) a SiµC+SiNWs chips. Right panels show the respective EDX spectra. White encircled areas show the spots where the EDX analysis was performed. SEM images revealed nanowires in the cytosol (white arrow). Counts per second (CPS). Scale bar = 1 µm.

We also imaged chip localizations inside HeLa cells. For such study, 150 nm-thick sections of resin-infiltrated cells were sliced by a microtome (see Experimental Section). SEM inspection and EDX analysis were used to confirm the presence of silicon inside the cells (Figure 5). Our previous works demonstrated that SiµCs could be found in tight association with endosomal membranes (Figure 5a) [33]. On the contrary, images of internalized SiµC+SiNWs and SiNW-Meshes suggested that they could be encircled by a lax endosomal-like membrane (Figure 5b and Figure 3c right panel). Cell membrane piercing by nanowires has been previously reported on cells laying on silicon nanowire substrates [7,34,35,36]. Although, from our images, it seemed that some silicon nanowires pierced the endosomal membrane and reached the cytosol (Figure 5b), we could not confirm this by EDX analysis, as the nanowires could be wrapped by a tight endosomal membrane impossible to resolve by this technique. Thus, chemical modification of the silicon nanowires with fluorescence dyes, which was beyond the scope of this work, will be required to undoubtedly demonstrate the ability of these nanowires to reach the cytosol [37].

## 4. Discussion

In this work, the foundational technology used to fabricate SiµCs [11] was extrapolated to develop distinct microchips decorated with silicon nanowires (SiµC+SiNWs) and isolated silicon nanowire entangled meshes (SiNW-Meshes). The CVD process that defined the polysilicon thickness was tuned to obtain either a 500 nm-thick layer as a base of SiµC+SiNWs or a layer of polysilicon nanoclusters for SiNW-Meshes. Gold nanoparticles were then selectively deposited over the polysilicon portions of both substrates to provide an anchoring position for subsequent silicon nanowire growth. An accurate characterization of the silicon nanowires using SEM allowed the determination of their width, length, density, and distribution in both cases (Table 1). Importantly, the width of the nanowires grown over the chips or nanoclusters batches was comparable, demonstrating a consistent growth mechanism throughout the samples. Furthermore, the increased length of the nanowires in the entangled meshes was due to a longer growing time, as it was necessary to obtain nanowires long enough to assure the robustness of these particles due to their extreme fragility resulting from their lack of support, instead provided to SiµCs-NWs by the 500 nm-thick polysilicon layer. Thus, the release and collection method had to be modified to avoid breaking the devices.

Further on, the interaction between cells and microchips was studied. Initially, cell viability assays in HeLa cells demonstrated that the cells with and without internalized silicon chips had the same viability, providing robust evidence that neither the material nor the structure or the chips altered the functionality of the cells. Likewise, the internalization capability was similar for the three different morphologies. Nonetheless, a morphological dependence in the initial stages of chip internalization was observed. Both SiµCs and the SiNW-Meshes, with comparable top and bottom faces, were internalized into the living cells top-side-up and bottom-side-up at similar rates. Alternatively, the very different morphological faces of SiµC-NWs presented two possible internalization pathways. It is also worth mentioning that SiNW-Meshes did not present statistical differences in chip orientation or number of internalized chips, suggesting that the remaining gold nanoparticles at the tip of the nanowires did not interfere or affect the internalization process [38]. Overall, the observed results suggest that HeLa cells prefer specific interfaces in the first stages of cell internalization. This observation is in good agreement with other works focused on 3D nanostructured platforms for cell trapping, which enhanced local topographic interactions with extracellular extensions [24,25]. The inspection showed that SiNWs share nanoscaled size and shape with cellular surface components (Figure S6) like filopodia [39], suggesting that bio-inspired nanomaterials could mimic cell structures and could be used to obtain new tools for cell biology. To rule out possible interferences in the final intracellular position of the chips due to their initial position, sedimentation studies of SiµCs and SiNW-Mesh were performed and showed that there was not a preferred chip direction when the particles settled down. The fact that SiµC+SiNWs were preferentially deposited with their micropart in a lower position, while their nanowire side appeared in contact with the cell membrane (Figure 4b) clearly supports the proposition that HeLa cells prefer the silicon nanowires at the initial stage of chip internalization. The percentage of chips inside the cells was similar for all the types of chips (Figure 3e). Previous works report that particles of different shape and size could be internalized by different pathways [40], and cellular uptake kinetics could depend on particle size [32]. These ideas along with the fact that, before internalization, the cells constantly manipulate the chips, allowing the contact of their micro- and the nanoparts with the cell membrane, could enhance the probability that cell internalize the chips by the most favorable pathway, in this case, that involving the nanoparts.

Lastly, EDX was used for material identification (Figure 4f and Figure S5 and Materials and Methods), allowing us to track to final location and interactions of the chips inside the cells. Interestingly, the malleable membrane surrounding the chips could be pierced by physical means by the nanowires, suggesting a direct passage from the endosomal membrane to the cytosol. Further chemical modification of the nanowires could provide the means to corroborate and exploit the endosomal–cytosol orifice, opening new opportunities for intracellular applications like drug delivery, sensing applications for biomolecular recognition, or nanomechanical sensors.

## 5. Conclusions

We have developed a technology which allows a batch production of nanowire silicon chips with controlled dimensions and that are cell-internalizable. In addition, these devices can be collected and suspended for their posterior use in cell cultures. Cell viability assays with micro- and nanowire chips were performed in HeLa cells and did not reveal any significant differences in viability when compared with control cells. Understanding how the geometry and size of a material affect the cell internalization processes is a crucial issue for the development of future intracellular tools. Hence, in this work, we fabricated nanowire chips entirely made of a unique, fluorescence dye-free material, i.e., silicon, and determined the effects of morphology and size on cell internalization. Our experiments showed that the internalization ratio for silicon chips with integrated SiNWs and SiNWs meshes was similar to that for non-nanostructured chips. However, differences were revealed in the initial stage of chip internalization. HeLa cells prefer SiNWs sides (nano- instead of microstructured silicon) for initial uptake. This could be related to the fact that particles of different size could be internalized by different pathways with different kinetics [35]. Finally, the ability to produce chips decorated with a large amount of silicon nanowires and with a high area-to-volume ratio that allows a large payload opens new opportunities for intracellular drug delivery. Furthermore, chips trapped in lax membrane-bound compartments can provide an enhanced loading capacity [38] and could be released in the cytosol by membrane disruption. Additionally, nanowire intracellular devices have a potential use in sensing applications [7,39]. For instance, chemical functionalization of the nanowires could produce valuable devices for biomolecular recognition [23,32]. In addition, the implementation of SiNWs on intracellular chips as nanomechanical sensors [40] will provide invaluable information on intracellular forces involved in many fundamental cellular processes. In conclusion, the capabilities of the cell-internalizable nanowire silicon chips make them excellent candidates for future applications in living cells [41].

## Figures and Tables

**Table 1:** Morphological statistics of the polysilicon nanoclusters and silicon nanowires. The table shows the distribution of polysilicon nanocluster diameters (ø) and densities and of nanowire length, width, and density obtained for SiµC+SiNWs and SiNW-Meshes. Counts, n: polysilicon nanoclusters n = 6000, nanowires on SiµC+SiNWs n = 1500, and nanowires on SiNW-Meshes n = 2300.

		Ø (nm)	Density (clusters/µm^2^)
Polysilicon nanoclusters		65 ± 12	128 ± 8
	**Length** **(nm)**	**Width** **(nm)**	**Density** **(NWs/µm^2^)**
SiµC+SiNWs	885 ± 231	60 ± 19	28 ± 2
SiNW-Mesh	1124 ± 405	71 ± 16	47 ± 2

## Supplementary Materials

The following are available online at https://www.mdpi.com/2079-4991/10/5/893/s1, Figure S1: Detailed sequence of the steps for the fabrication of the silicon chips. Figure S2: Polysilicon film formation: Nucleation and growth. Figure S3: Silicon nanowires and polysilicon nanoclusters morphology. Figure S4: Nanowire length controlled by growth time. Figure S5: BFOM, SEM, and FIB inspection of cells and silicon chips. Figure S6: Silicon nanowires mimic cell surface structures.
