# Supplementary material for: Internalization and Viability Studies of Suspended Nanowire Silicon Chips in HeLa Cells
Source: Nanomaterials (Basel). 2020 May 7;10(5):893. doi: 10.3390/nano10050893 (PMC7279308; doi:10.3390/nano10050893)
Supplement: Supplementary file 1 [file nanomaterials-10-00893-s001.pdf]

nanomaterials

Supplementary Materials

Internalization and Viability Studies of Suspended Nanowire Silicon Chips in HeLa Cells

Sara Durán [1], Marta Duch [1], Rodrigo Gómez-Martínez [1], Marta Fernández-Regúlez [1], Juan Pablo Agusil [1], Manuel Reina [2], Claudia Müller [2], Álvaro San Paulo [3], Jaume Esteve [1], Susana Castel [2] and José A. Plaza [1,*]

[1] Instituto de Microelectrónica de Barcelona, IMB-CNM (CSIC), Campus UAB, Cerdanyola, 08193 Barcelona, Spain; sara.duran@imb-cnm.csic.es (S.D.); marta.duch@imb-cnm.csic.es (M.D.); rogomez@uniandes.edu.co (R.G.-M.); marta.fernandez@imb-cnm.csic.es (M.F.-R.); juanpablo.agusil@imb-cnm.csic.es (J.P.A.); jaume.esteve@imb-cnm.csic.es (J.E.)

[2] Departamento de Biología Celular, Fisiología e Inmunología, Facultad de Biología, Universitat de Barcelona, 08028 Barcelona, Spain; mreina@ub.edu (M.R.); cmuller@ub.edu (C.M.); scastel@ub.edu (S.C.)

[3] Instituto de Microelectrónica de Madrid, IMM-CNM (CSIC), Isaac Newton 8, Tres Cantos, 28760 Madrid, Spain; alvaro.sanpaulo@csic.es

* Correspondence: joseantonio.plaza@imb-cnm.csic.es; Tel.: +34-935-94-77-00

Received: 31 March 2020; Accepted: 3 May 2020; Published: 7 May 2020

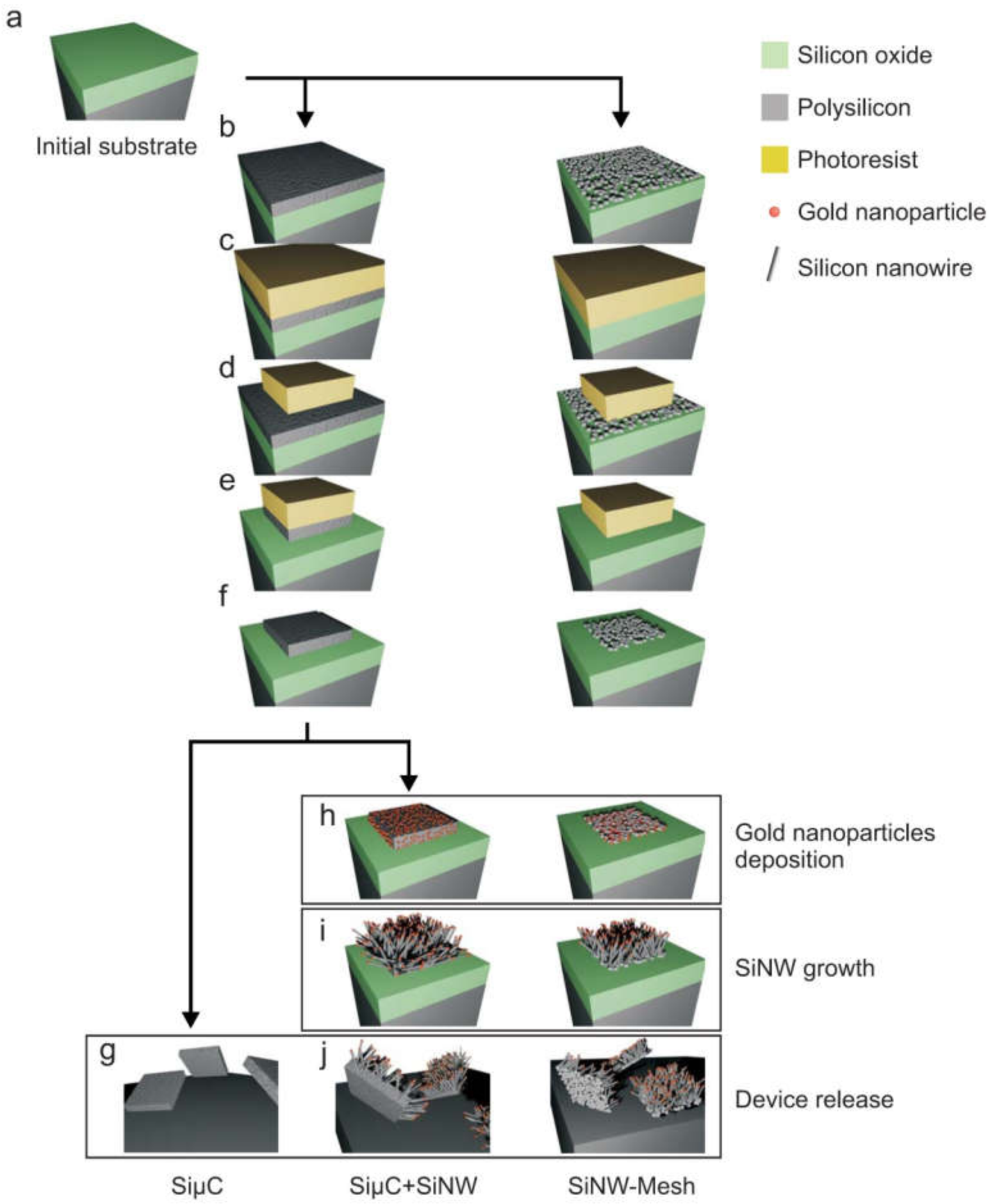

Figure S1. Detailed sequence of steps for the fabrication of the silicon chips. 100 mm Ø 500 μm-thick P-type <100> silicon wafers (Okmetic) were used as initial substrates. (**a**) A 1000 nm silicon oxide layer was grown by wet oxidation as sacrificial layer; (**b**) Left panel: A 500 nm-thick polysilicon layer was deposited by chemical vapor deposition to define the polysilicon-microchip thickness. Right panel: The polysilicon layer deposition was aborted at the nucleation stage forming polysilicon nanoclusters; (**c**) A 1.2 μm-thick positive photoresist was spun onto the wafers; (**d**) Exposure to UV light was performed through the first reticule. The photolithographic step defined the lateral dimensions of the chips (3 μm × 3 μm); (**e**) Etching away the exposed polysilicon defined the engineered particles; (**f**) Photoresist stripping. SiμCs fabrication; (**g**) The sacrificial etching in 49% HF vapors released the SiμCs from the wafer. SiμC+SiNWs and SiNW- meshes fabrication; (**h**) A galvanic displacement deposition of gold nanoparticles over the SiμC (Left panel) and polysilicon nanocluster (Right panel); (**i**) Silicon nanowires were grown by the VLS method; (**j**) The sacrificial etching in 49% HF vapors released the SiμC+SiNWs (Left panel) and the SiNW-Meshes (Right panel) from the wafer

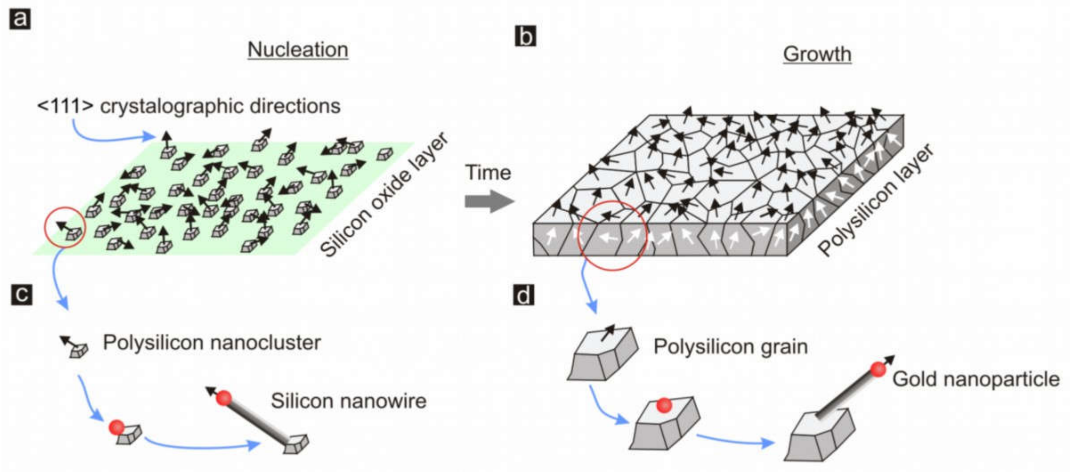

Figure S2. Polysilicon film formation: Nucleation and growth. (**a**) The formation of small clusters through the agglomeration of adsorbed silicon atoms occurs at the initial stages of the deposition process. Polysilicon nanoclusters have a random crystallographic orientation; (**b**) The enlargement of these nuclei forms a continuous film during the growth process. This film has small grains which also have a random crystallographic orientation; (**c**, **d**) The random orientation of the nanoclusters and grains induces a random growth of the silicon nanowires.

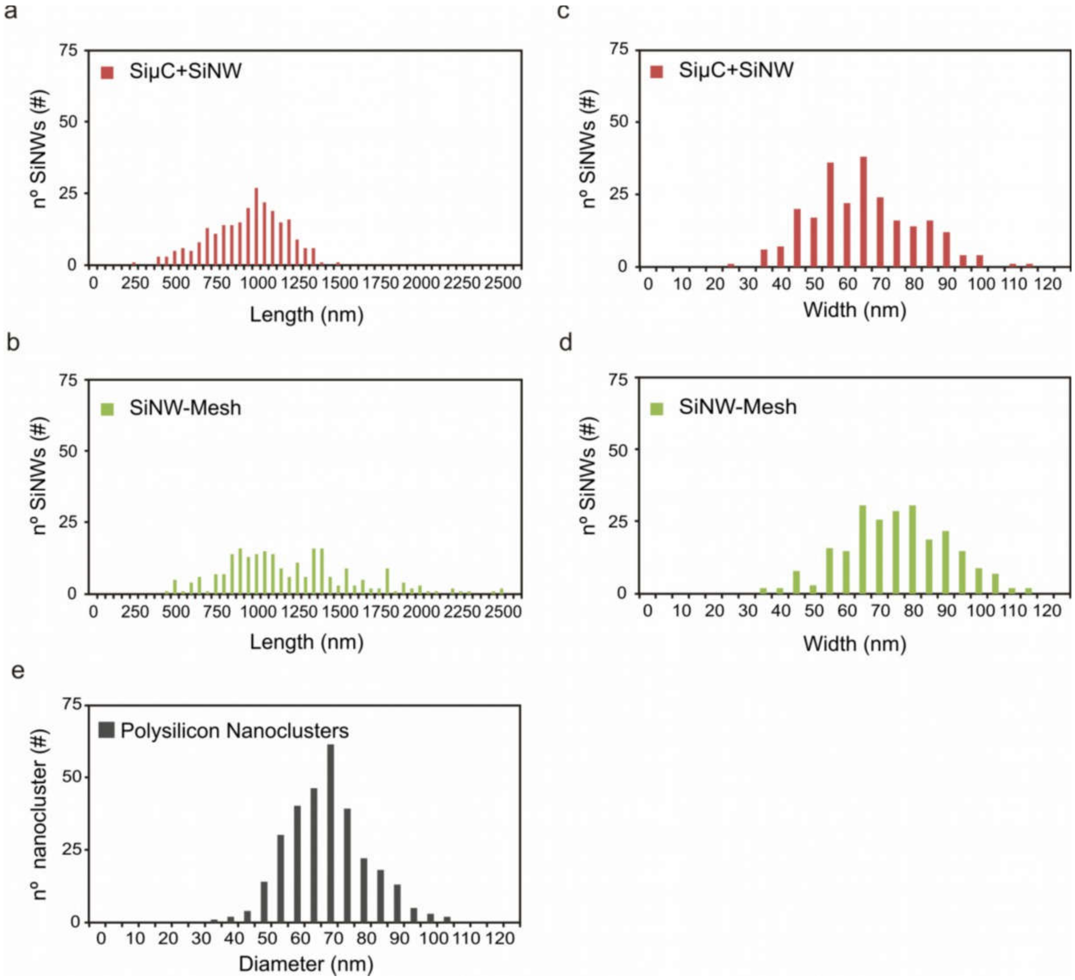

Figure S3. Silicon nanowires and polysilicon nanoclusters morphology. The plots show the distribution of nanowire (**a**, **b**) length, (**c**, **d**) width and (**e**) polysilicon nanocluster diameter obtained from the SiμC+SiNWs and SiNW-Meshes. Polysilicon nanoclusters n = 6000, SiμC+SiNWs n = 1500 and SiNW-Mesh n = 2300.

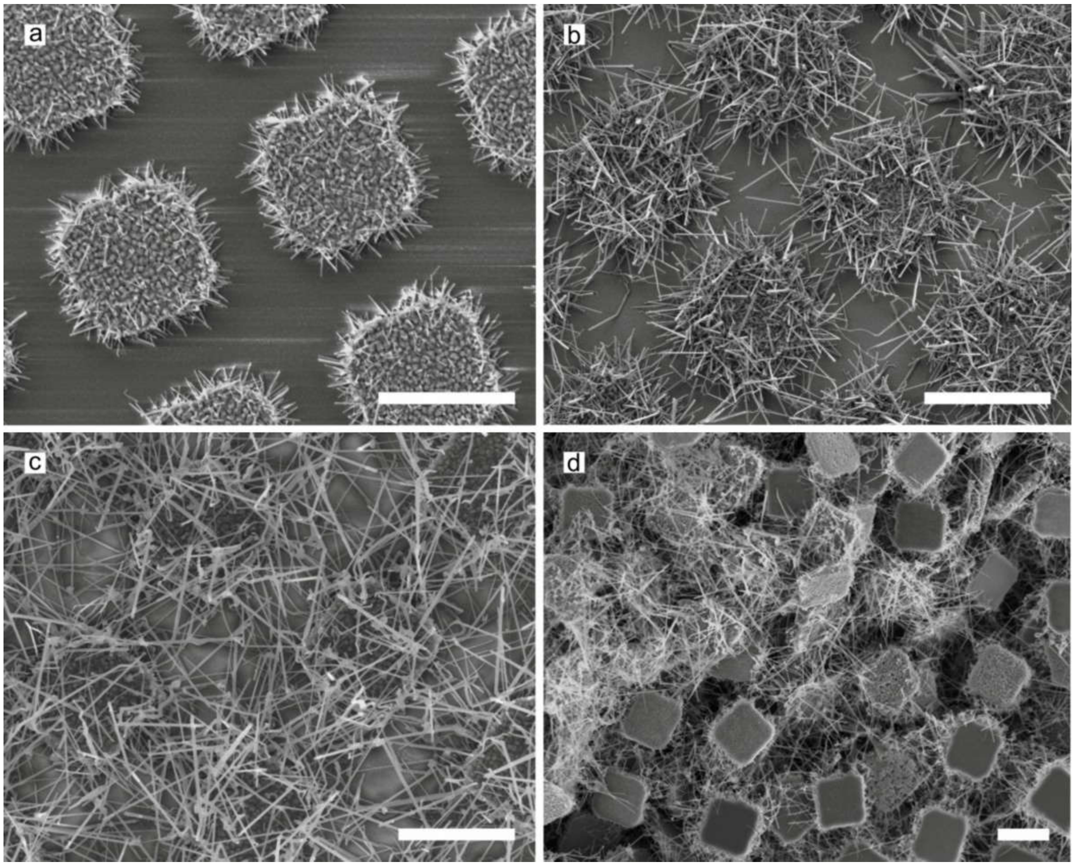

Figure S4. Nanowire length controlled by growth time**.** (**a**–**c**) Images show SiμCs decorated with silicon nanowires grown during 45 s, 60 s and 90 s, respectively; (**d**) Longer growthtimes created nanowires entanglements between neighboring chips which ruined the device collection. Scale bars = 3 μm.

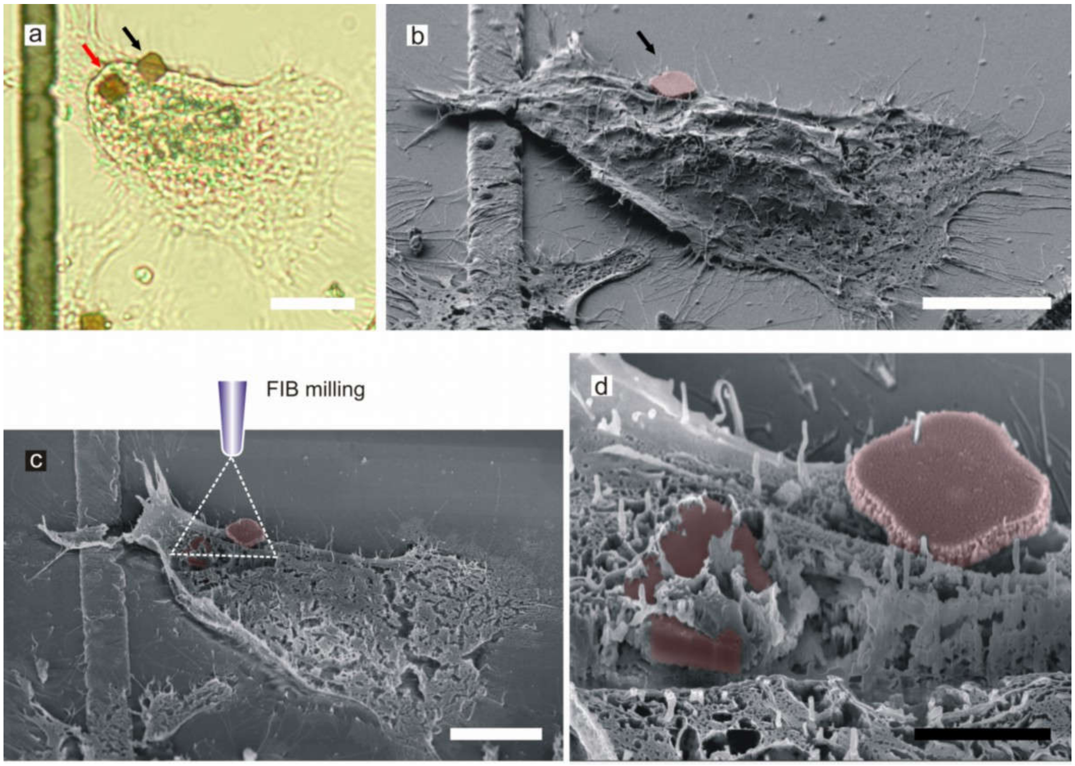

Figure S5. BFOM, SEM and FIB inspection of cells and silicon chips. (**a**) Silicon chips were clearly identified on bright field optical microscope due the high reflectivity of the silicon. However, the localization of the chips inside (red arrow) or outside (black arrow) the cell cannot be determined; (**b**) On the contrary, SEM inspection of the same cell only showed the chip outside the cell because electrons cannot penetrate inside the membrane and hence be reflected from the inner parts of the cell; (**c**, **d**) FIB nanomachining was used to section the cell in order to demonstrate that the second chip was cell-internalized. White scale bars = 10 μm. Black scale bar = 3 μm.

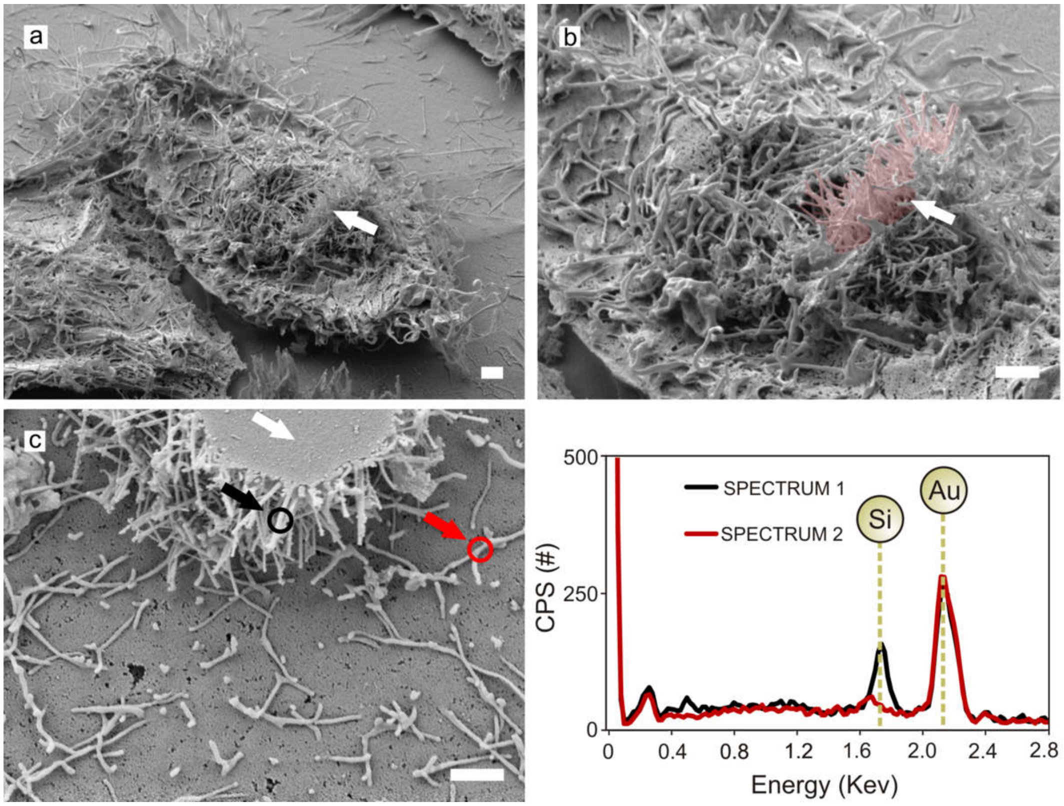

Figure S6. Silicon nanowires mimic cell surface structures. (**a**) SEM image of a HeLa cell with a semi-internalized SiNW-Mesh. The morphology of the silicon nanowires was visually indistinguishable from the cell filopodia; (**b**) Pseudo-colored zoom image of the same cell, which reveals the silicon nanowires (pink color); (**c**) Left panel shows a SEM image of a SiμC+SiNWs on a HeLa membrane where silicon nanowires are difficult to be distinguishable. Right panel: Energy-dispersive X-ray spectroscopy (EDX) was used to undoubtedly identify the presence of silicon. White arrows indicate chip localization. Red and black arrows indicate the selected filopodia and SiNW for the EDX analysis, respectively. Scale bar = 1 μm.

© 2020 by the authors. Submitted for possible open access publication under the terms and conditions of the Creative Commons Attribution (CC BY) license (http://creativecommons.org/licenses/by/4.0/).
